# A computational guided, functional validation of a novel therapeutic antibody proposes Notch signaling as a clinical relevant and druggable target in glioma

**DOI:** 10.1038/s41598-020-72480-y

**Published:** 2020-10-01

**Authors:** Dayana Herrera-Rios, Guanzhang Li, Dilaware Khan, Julia Tsiampali, Ann-Christin Nickel, Philippe Aretz, Michael Hewera, Abiagail Kora Suwala, Tao Jiang, Hans-Jakob Steiger, Marcel Alexander Kamp, Sajjad Muhammad, Daniel Hänggi, Jarek Maciaczyk, Wei Zhang, Ulf Dietrich Kahlert

**Affiliations:** 1 Neurosurgical Clinic, Medical Faculty, Heinrich-Heine University Duesseldorf, Moorenstrasse 5, 40225 Dusseldorf, Germany; 2 Beijing Neurosurgical Institute, Capital Medical University, Beijing, China; 3 Department of Neuropathology, University Hospital Heidelberg, Heidelberg, Germany; 4 Clinical Cooperation Unit Neuropathology, German Cancer Consortium (DKTK), Duesseldorf, Germany; 5 Department of Neurosurgery, University of Bonn, Bonn, Germany; 6 DKTK, German Cancer Consortium, Essen/Duesseldorf, Germany; 7 Present Address: Skin Cancer Unit of the Dermatology Department, Medical Faculty, West German Cancer Center, University Duisburg-Essen, 45147 Essen, Germany

**Keywords:** Cancer genetics, Cancer stem cells, CNS cancer, Cellular signalling networks, Data mining, Gene regulatory networks

## Abstract

The Notch signaling network determines stemness in various tissues and targeting signaling activity in malignant brain cancers by gamma-secretase inhibitors (GSI) has shown promising preclinical success. However, the clinical translation remains challenging due to severe toxicity side effects and emergence of therapy resistance. Better anti-Notch directed therapies, specifically directed against the tumor promoting Notch receptor 1 signaling framework, and biomarkers predicting response to such therapy are of highest clinical need. We assessed multiple patient datasets to probe the clinical relevance Notch1 activation and possible differential distribution amongst molecular subtypes in brain cancers. We functionally assessed the biological effects of the *first-in-human* tested blocking antibody against Notch1 receptor (brontictuzumab, BRON) in a collection of glioma stem-like cell (GSC) models and compared its effects to genetic Notch1 inhibition as well as classical pharmacological Notch inhibitor treatment using gamma-secretase inhibitor MRK003. We also assess effects on Wingless (WNT) stem cell signaling activation, which includes the interrogation of genetic WNT inhibition models. Our computed transcriptional Notch pathway activation score is upregulated in neural stem cells, as compared to astrocytes; as well as in GSCs, as compared to differentiated glioblastoma cells. Moreover, the Notch signature is clinical predictive in our glioblastoma patient discovery and validation cohort. Notch signature is significantly increased in tumors with mutant IDH1 genome and tumors without 1p and 19q co-deletion. In GSCs with elevated Notch1 expression, BRON treatment blocks transcription of Notch pathway target genes Hes1/Hey1, significantly reduced the amount of cleaved Notch1 receptor protein and caused significantly impairment of cellular invasion. Benchmarking this phenotype to those observed with genetic Notch1 inhibition in corresponding cell models did result in higher reduction of cell invasion under chemotherapy. BRON treatment caused signs of upregulation of Wingless (WNT) stem cell signaling activity, and vice versa, blockage of WNT signaling caused induction of Notch target gene expression in our models. We extend the list of evidences that elevated Notch signal expression is a biomarker signature declaring stem cell prevalence and useful for predicting negative clinical course in glioblastoma. By using functional assays, we validated a first in man tested Notch1 receptor specific antibody as a promising drug candidate in the context of neuro oncology and propose biomarker panel to predict resistance and therapy success of this treatment option. We note that the observed phenotype seems only in part due to Notch1 blockage and the drug candidate leads to activation of off target signals. Further studies addressing a possible emergence of therapy resistance due to WNT activation need to be conducted. We further validated our 3D disease modeling technology to be of benefit for drug development projects.

## Introduction

Glioblastoma, the most common primary malignant brain cancer in adults, accounts with a median survival time of less than 2 years to one of the most lethal cancers overall^1^. Despite recent high-profile work on single cell analytics of tumor cells showed inter- and intra-tumoral heterogeneity of the disease is explained by the existence of four consensus cellular stages^2^, the field still recognizes the existence of a stem cell-like cell population to promote the pathology of glioblastoma^3, 4^. Targeting aberrantly activated phylogenetically conserved stem cell signaling pathways is historically considered a powerful strategy to fight glioblastoma with several promising clinical trials underway^5, 6^. One very prominent, and in the context of glioblastoma heavily studied pathway is Notch signaling.

The Notch network consists of canonical and non-canonical branches. Canonical Notch is regulated through ligand interaction of four different types of pathway receptors (Notch1,2,3,4)^7^. Clinically most advanced anti-Notch chemotherapeutics are inhibitors of gamma-secretase, the enzyme responsible for the final pathway activating cleavage of the intracellular domains of the Notch receptors^6^. The contribution of the individual pathway receptors to the pathobiology of glioblastoma is not fully understood. Notch1 signaling has been studied the most revealing its high activation that is of use as a biomarker in precision neuro-oncology^8^ and promotes various mechanisms of glioblastoma malignancy such as cell survival^9^, growth^10^, motility^11, 12^ , immunogenicity^13^ and tumorigenicity^14^. Elevated Notch1 activation in glioblastoma is associated to micro-environmental regulated stem cell niches such as under hypoxia^15^ or the vascularization zone^16, 17^ and has been shown to promote glioma cell resistance to autophagy^18^ or radiation^19^. These strong indications of biological relevance and therapeutic potential of targeting Notch1 in glioblastoma, together with recent promising results on a new anti-Notch1 directed drug candidate^20^, the *first-in-man* tested humanized anti-Notch1 blocking antibody termed brontictuzumab (BRON), in other cancers^21–23^ encouraged us to study the biologic consequences of BRON on models systems of glioblastoma stem-like cells.

In our study, we applied computational biology on biobank data retrieved from various clinical cohorts and present a transcriptional Notch activity signature that identifies stem cell prevalence in neuronal context, both in glioblastoma and normal development. Moreover, we hypothesize this signature is useful to develop biomarker-guided diagnostics of brain cancer as its high activation predicts for negative clinical course of the patient and associates to certain DNA status of the tumor used for clinical stratification of the disease. Our functional studies found that high Notch1 expression predicts for glioblastoma cell sensitivity to BRON as measured by inhibition of Notch target genes Hes1/Hey1 and reduction of cleaved Notch1 receptor protein. BRON exhibits superior effect on inhibiting glioblastoma cell invasion as benchmarked to gamma-secretase inhibitor (GSI) treatment or genetic inhibition of Notch1 respectively. Off-target effects of BRON treatment, such as though activation WNT signaling in GSCs, possibly contributing to the blockade of cell invasion or possibly causing emergence of therapy resistance, need to be addressed in follow up studies in order to validate the translational utility of this drug candidate in the context of neuro oncology.

## Methods

### Sample and database

This study was approved by Capital Medical University Institutional Review Board (IRB). In total, 325 glioma patients with transcriptome sequencing data were enrolled in the discovery cohort and 693 glioma patients with transcriptome sequencing data were enrolled in the validation cohort. The transcriptome sequencing data was originally generated by Agilent Whole Human Genome Array platform. Molecular testing of each patient was performed at the Molecular Pathology Testing Center of Beijing Neurosurgical Institute. The sequencing data, clinical information of glioma patients was uploaded to the CGGA portal (https://cgga.org.cn/). The detailed patient information is shown in Table S1. Transcriptome microarray data of human astrocyte cultures, one source of human neural stem cells (NSC 16WF), glioblastoma stem-like cells (GSCs) and differentiated counterparts were obtained from GSE67089^24^.

### Computational analysis

The biological functional enrichment score of each patient was generated by Gene Set Variation Analysis (GSVA) analysis using the default parameters by the gsva package in R. GSVA is a widely applied, and generally accepted scientific technique for characterizing activation of pathways from gene expression dataset^25^.

Gene list of each terms was downloaded from Kyoto Encyclopedia of Genes and Genomes (KEGG) Web portals (https://www.kegg.jp/)^26^.

Significance of correlation of Notch score activity to the expression level of consensus genes of the Notch pathway is based on the calculation coefficient R-value as obtained by *pearson* correlation analysis.

Differences in Notch scores of patients in different molecular feature were tested by two-tailed Student’s t-test. The detailed results summarized in Fig. 3 are shown in Supplementary Figure S3.

To perform protein network analysis, Notch and WNT related genes as downloaded from GSEA index (in May 2020) were uploaded to STRING software tool (https://string-db.org).

### Cell culture, DNA constructs and viral infection

GBM cells were generously provided by A. Vescovi, Milan, Italy (GBM1); MS Carro, Freiburg, Germany (BTSC407/ 407p), A. Weyerbrock, Freiburg, Germany (U87), G. Riggins, Baltimore, USA (JHH520 / JHH) and EH Raabe, Johns Hopkins Baltimore (SF188). All cells were cultured as neurospheres (NS) in serum-free and glutamine-rich conditions as described before and passaged at least twice per week^27^. All cells were regularly characterized for myco-plasma negativity and cell line authenticity was assessed as previously described^28^. All cell models expressed wildtype form of isocytrate dehydrogenase 1. Studies are conducted under measures of our GLP-inspired quality control system^29^. 

The following lentiviral constructs were used: empty control pLKO.1 TCR cloning vector (Addgene plasmid #10878^30^) as well as pLKO.1 vector with cloned interference RNA constructs directed against Notch1 mRNA as previously described^31^. Canonical WNT pathway activity was assessed using bioluminescence-based quantification with luciferase reporter construct (firefly luciferase cassette under the control of 7 (T-Cell factor) TCF binding sites, as previously described^32^. Luciferase signals were detected with Dual-Light Luciferase & β-galactosidase reporter gene assay (Thermo Fisher, USA) similarly as previously described^33^. Constitutive blockade of canonical WNT was achieved by genetic means through stable expression of shRNA cocktail targeting CTNNB1 as previously described^27^.

Lentiviral particles were produced by transfecting 293 T cells with the lentiviral packaging system using GeneJuice (Merck Millipore) per the manufacturer’s instructions as previously described^34^. Supernatants were collected 48 and 72 h post transfection and passed through a 0.45-micron filter before concentrated using 50% polyethylene glycol and NaCl and then frozen at − 80 °C until needed^34^.

### DNA mutation analysis

DNA mutation analysis was performed according to Zacher et al*.*^35^. Briefly, DNA extraction from frozen cell samples was performed using Maxwell RSC Blood DNA Kit (Promega, AS1400) and quantified using the QuantiFluor ONE dsDNA System (Promega, E4871) followed by TaqMan RNase P Detection Reagents Kit (Life Technologies) using a StepOne Plus real‐time PCR machine (Life Technologies). Library preparation were generated using the Ion AmpliSeq Library 2.0 Kit (Life Technologies) and the customized AmpliSeq glioma gene panel. Sequencing was performed on Ion S5 (Life Technologies) and aligned to the human reference genome GRC37 (hg19) using the Torrent Suite 5.12.1.0 software. The sequence and copy number variants analysis was performed using the software Ion Reporter v5.12.0.0, IGV v.2.5.0 and NextGene Version 2.4.2.2.

### Determination of IC_50_

In this assay we tested a concentration range of brontictuzumab up to 50 µg/ml for 48 h. The concentration that resulted in about 50% reduction of expression of Notch target genes HES1 and HEY1 was defined as IC_50_. Cells designed as non-responder did not react with target suppression. Since IC_50_ of MRK003 in some of the cell models was reproducibly established in previous studies ^28^, we used those *minimum drug consensus concentrations of* 0.5 µM and 1 µM, a drug range *generally considered clinical relevant and achievable in patients serum.*

### mRNA and protein analyses

The abundance of mRNA was assessed by conversion into complementary DNA and subsequent quantitative real-time PCR measurements using SYBR green-based fluorescence (Bio-Rad, USA). Relative quantification to housekeeping gene β-actin (ACTINB) was assessed with ddCt-method. Primers were obtained through Sigma- Aldrich Germany and respective sequences can be found in Suppl. Figure 1.

Western blotting was performed as described before^34^, antibodies were used as per the manufacturer’s instructions (specifications see Suppl. Figure 1). Total protein abundance was determined colorimetrically using DC Protein assay (Biorad) and densitometry of the transferred proteins was done electronically as previously described^36^.

### In vitro* growth* and invasion assay

*Cellular growth of glioblastoma neurospheres was assessed using CellTiter Blue Viability assay (Promega, USA) similarly as described before*^36^ *plating 2.000cells/ well in 100 µl growth media. For each growth study one representative curve is displayed in the figures.*

Assessment of cellular invasion was performed using modified 24 well Boyden Chamber assay similarly as described before^34^.The inserts were coated with growth factor reduced Matrigel (Becton Dickinson Biosciences, USA) and incubated for 1 h at 37 °C. Subsequently, 75.000 cells suspended in 500 μl DMEM (Life Technologies, USA) were placed on top of each insert membrane. The bottom was filled with 800 μl DMEM media containing 10% fetal calf serum. All Boyden chamber assays were analyzed 20 h after cell plating. The upper side of the membrane was then wiped carefully with a cotton swab to remove the rest of the plated cells. The membrane was then fixed in ice cold methanol for 10 min and stained with Hematoxylin for 5 min. The invasion of the cells was evaluated by counting the cell nuclei on the lower side of the membrane under a light microscope (5 random high power fields/ insert).

Antibody invasiveness’ effect was tested after seven days. The first day cells were enzymatic treated to have single cells. During next six days, cells were in culture under treatment, every 2 days media was completely renewed. On day six, the invasion assay was performed as mentioned above. For MRK003 treatment, invasion assay was carried out after 20 h in total.

### Protein interaction network analysis

The interaction between proteins in Notch and WNT pathways was analyzed in the STRING (v11.0, https://string-db.org/cgi). The protein interaction network was visualized with Cytoscape (v3.7.2) as described in the previous study^37^.

### Reporting experiments on the use of human tissue samples

All methods were carried out in accordance with relevant guidelines and regulations. All experimental protocols were approved by the ethical commission of the medical faculty of the Heinrich-Heine-University Duesseldorf, Germany. An informed consent was obtained from all patients donated samples that contributed to the study results (from Capital Medical University Beijing).

### Statistical analyses

If not mentioned otherwise, experiments were performed in n = 3 independent repetitions. Statistical analyses were performed using Student’s t-test and presented as mean values plus standard deviation if not indicated otherwise. **p = 0.05, *** p = 0.001, **** p = 0.0001.

### Ethical approval

All studies were approved if relevant: The reported study using CGGA data was approved by the Beijing Tiantan Hospital institutional review board and tumor specimen quality control. The use of cell models to study genes involved in brain cancer biology was approved by the ethical commission of the medical faculty of Heinrich-Heine University (study ID 5841R).

## Results

### Establishment of Notch activation signature in clinical samples of glioblastoma

By interrogating CGGA sequencing data, we calculated a transcriptional Notch pathway activation signature using GSVA. GSVA is a technique for characterizing activation of pathways from gene expression dataset^25^ and is commonly used to identify expression enrichments between different samples/ group of samples^38–41^.

**Figure 1:**
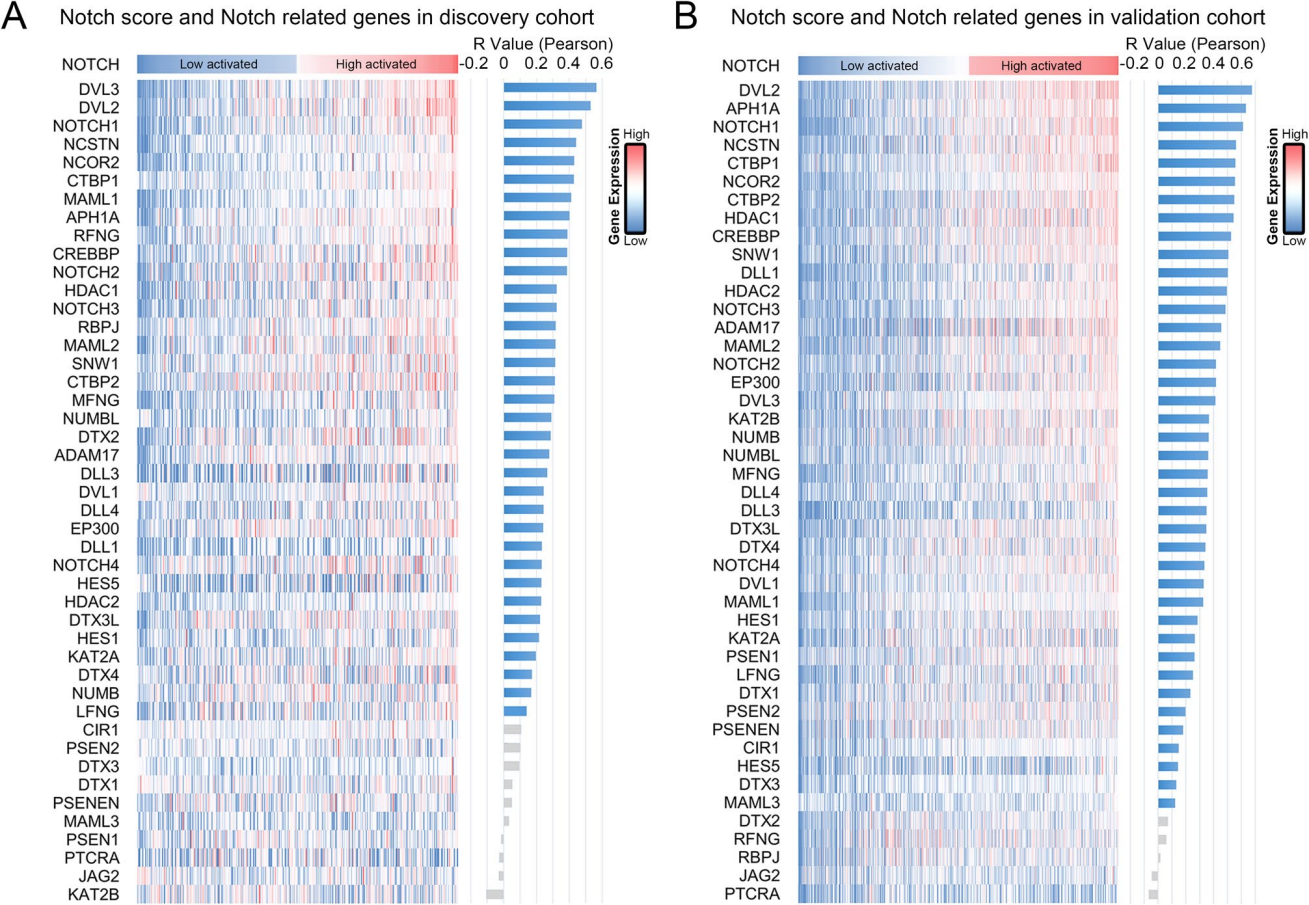
Notch pathway activation in our clinical samples can be represented by Notch score. (**A** and **B**) Heatmap of our computational analysis in our patient data shows that our calculated Notch activation score correlates with gene expression of many consensus Notch pathway members. Blue columns represented significant correlation and grey ones represented no significant correlation.

In order to verify the credibility of the Notch score, correlation analysis was performed between our enrichment calculation and consensus Notch signaling genes. In discovery cohort (325 patients), 35 of 45 genes associated with the Notch pathway showed significant positive correlation with our Notch score (Fig. 1A). Similarly, in the validation cohort (693 glioma patients), the Notch score was significantly positively correlated with most consensus Notch genes (40 of 45 genes) (Fig. 1B). In both cohorts, Notch1 ranks the third most enriched gene. Our computational results propose an approach to develop a biomarker signature to determine Notch pathway activation status in clinical samples. Details on Notch score calculation results can be found in Table S2.

### Notch pathway is increased in stem cell populations and possesses predictive value for the clinical course of brain cancer patients

**Figure 2:**
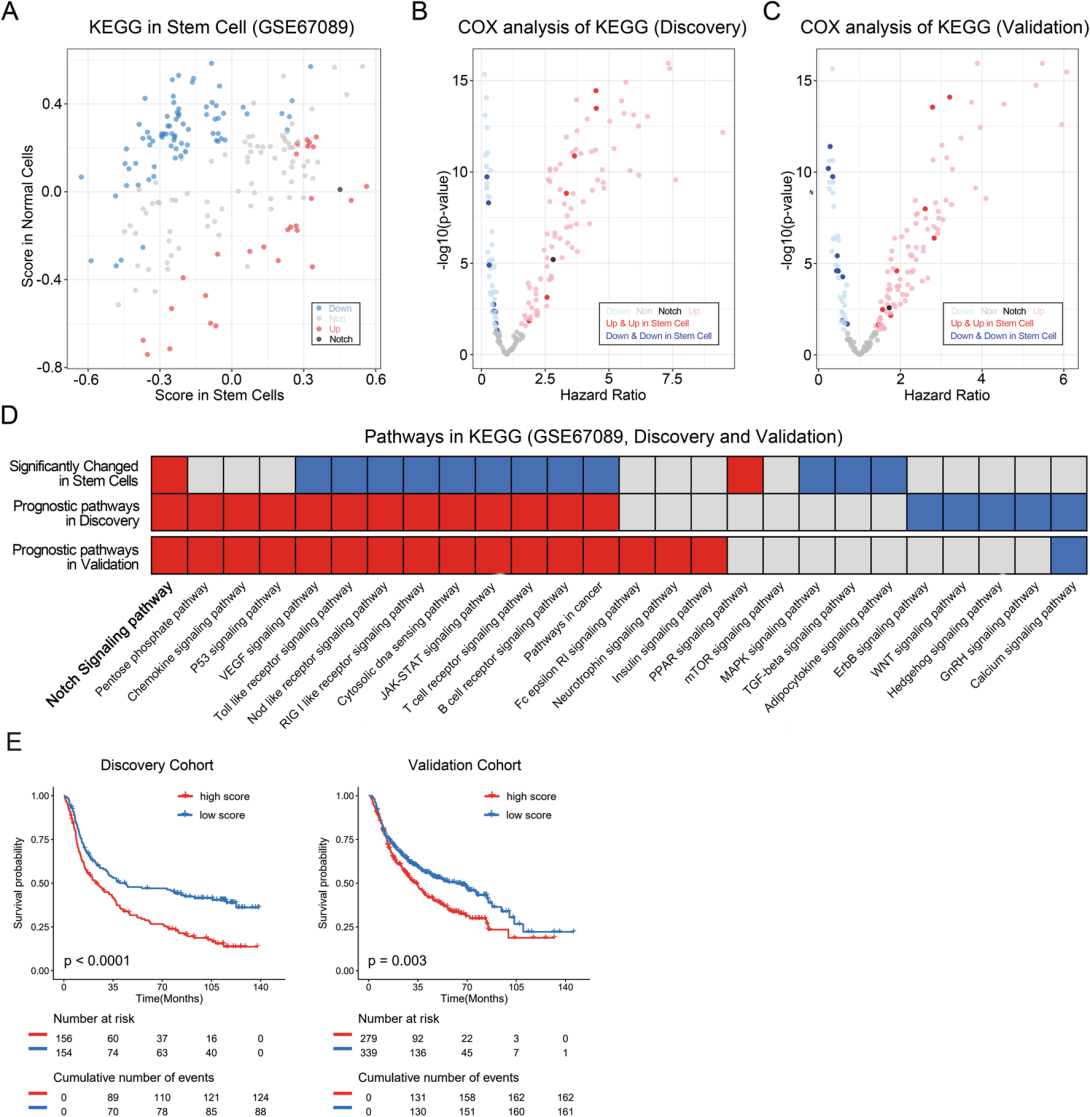
Notch pathway is activated in neural stem cells and associated with poor prognosis. (**A**) Enrichment scores of 28 terms significantly enriched in neural stem cells as compared to astrocytes (red and black dots), whereas 76 terms are significantly enriched in astrocytes (blue dots). Grey dots represented no significant difference between astrocytes and fetal neural stem cells. (**B** and **C**) Volcano chart showed the prognostic value of KEGG terms in discovery and validation cohorts. Dark blue dots represent KEGG terms that are highly enriched in astrocytes with positive prognostic value in glioma patients. Light blue dots represent KEGG terms that are not enriched in astrocytes but possess positive prognostic value. Dark red and black dots represent KEGG terms that are highly enriched in neural stem cells with poor prognostic value in glioma patients. Light red dots represent KEGG terms that are not enriched in neural stem cells but possess positive prognostic value. (**D**) The heatmap showed whether cancer related pathways are enriched of in neural stem cells and whether prognostic significance of pathways in glioma patients exists. Red squares represent pathways highly enriched in neural stem cells or with poor prognostic value. Blue squares represent pathways highly enriched in astrocytes or with better prognostic value. Grey ones represent no significant enriched or without prognostic value. (**E**) Overall survival probability chart of patients in discovery and validation cohort in dependency of Notch activation status in their tumors.

To understand the stem cell-specific activation of given pathways, the enrichment score of accepted 186 KEGG terms were calculated and their abundancy in the transcriptome of human astrocytes was compared to those in neural stem cells. In neural stem cells, 28 KEEG terms are significantly increased and 76 terms are significantly decreased (Fig. 2A). The prognostic significance of the KEGG terms was explored in our patient discovery and validation databases (Fig. 2B,C). We further performed an enrichment analysis of consensus KEGG items annotated to cancer related signaling pathways (Fig. 2D). Out of all those analysis, only Notch pathway is significantly activated in neural stem cells and possess significant clinical prognostic value in discovery and validation databases. Concordantly, high Notch score in our clinical samples is significantly negative prognostic in terms of median overall survival of the patients (Fig. 2E).

We further performed KEGG item comparison of glioblastoma stem cells and differentiated counterparts. Although not significant, Notch pathway is enriched in stem cell models (p-value = 0.07; Table S5). Supplementary tables presenting details KEGG items that are upregulated in neural stem cells (Suppl. Table S3), KEGG items that are clinical prognostic (Suppl. Table S4) and KEGG items that are differentially regulated in glioma stem cells versus differentiated counterparts (Suppl. Table S5).

### Notch activation associates to clinical diagnostic markers in glioblastoma

**Figure 3:**
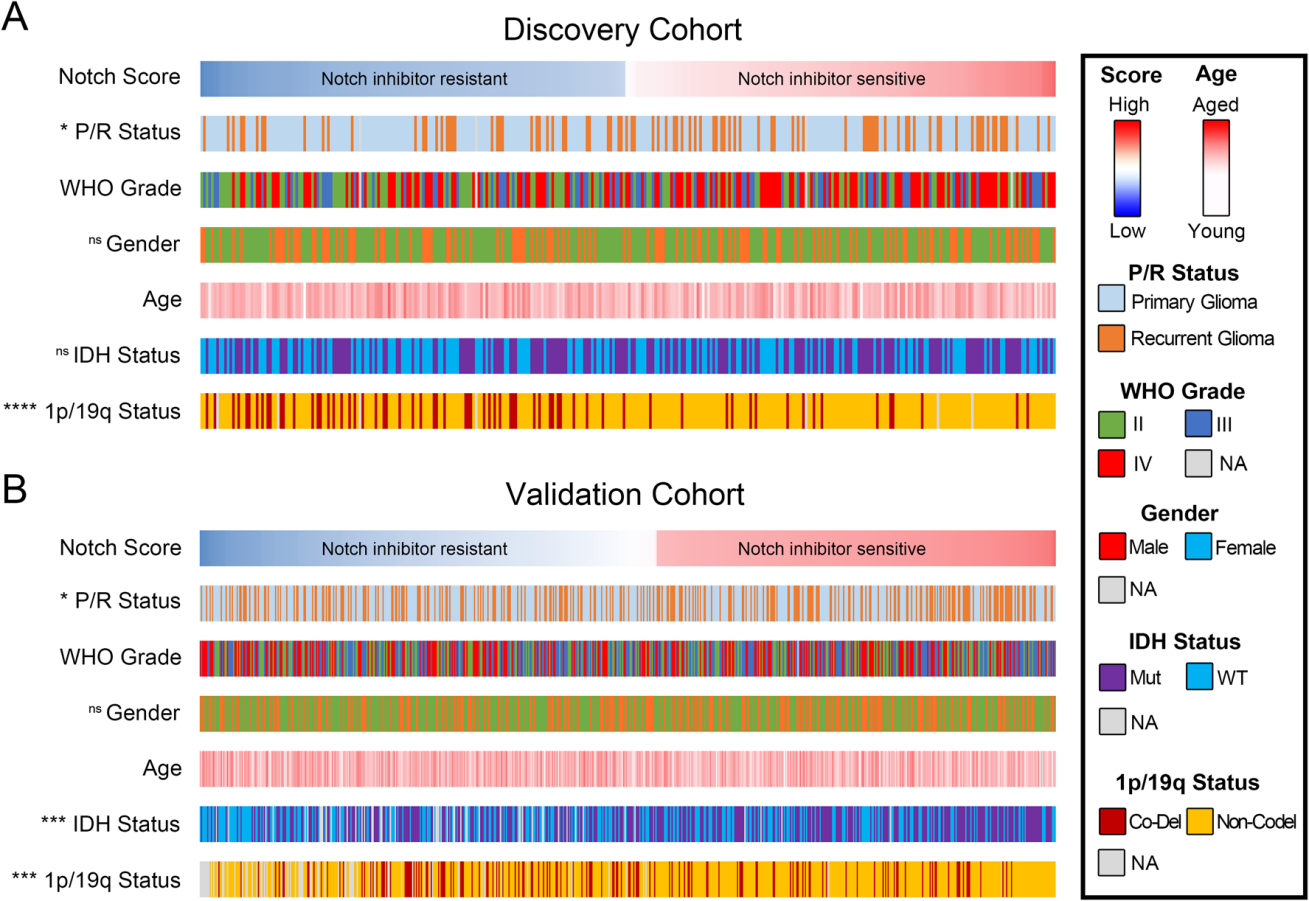
Notch expression associates to molecular properties of gliomas. (**A** and **B**) Notch score was significantly increased in recurrent gliomas, in tumors with mutant form of IDH1 and in cases without chromosome 1p/19q co-deletion. The significance of the difference between two groups was assessed by student's t test, between three groups by one-way ANOVA. Numbers of each cases associated to molecular subtype are provided in Supplementary File S6.

Given previous findings of high Notch activation status in glioblastoma cells to be predictive for Notch-inhibition therapy success^42^, we termed our clinical samples with low Notch activation into “Notch inhibitor sensitive” and samples with high Notch activation in “Notch inhibitor resistant” cases. We found recurrent glioma patients with R132H-mutated DNA of the gene isocytrate dehydrogenase 1 (IDH1) and patients without chromosome 1p/19q co-deletion were most likely sensitive to Notch inhibitors. Sensitivity to Notch inhibitors was independent of gender, age, and tumor WHO grade of patients with glioma (Fig. 3A,B). There was no difference in mutational burden in the tumors with high and low Notch activation score (Suppl. Figure S5).

In the next step we aimed to validate our in silico data with functional models. Given the challenges of culturing the field experiences regarding the availability of cell models with IDH1R132H or 1p/19q co-deletion, we focused our analysis on consensus Notch pathway genes, which are most strongly associated to our clinical Notch activation score. Given the importance of Notch1 in pathway regulation and in the biology of glioma, out of the top genes we chose Notch1 and tested a novel Notch1-targeting clinical drug candidate on stem cell models of glioblastoma.

### Elevated Notch1 transcription predicts sensitivity to brontictuzumab that can be monitored by Hes1/Hey mRNA inhibition and reduction of cleaved Notch1 receptor, not associated to wild type p53 status

**Figure 4:**
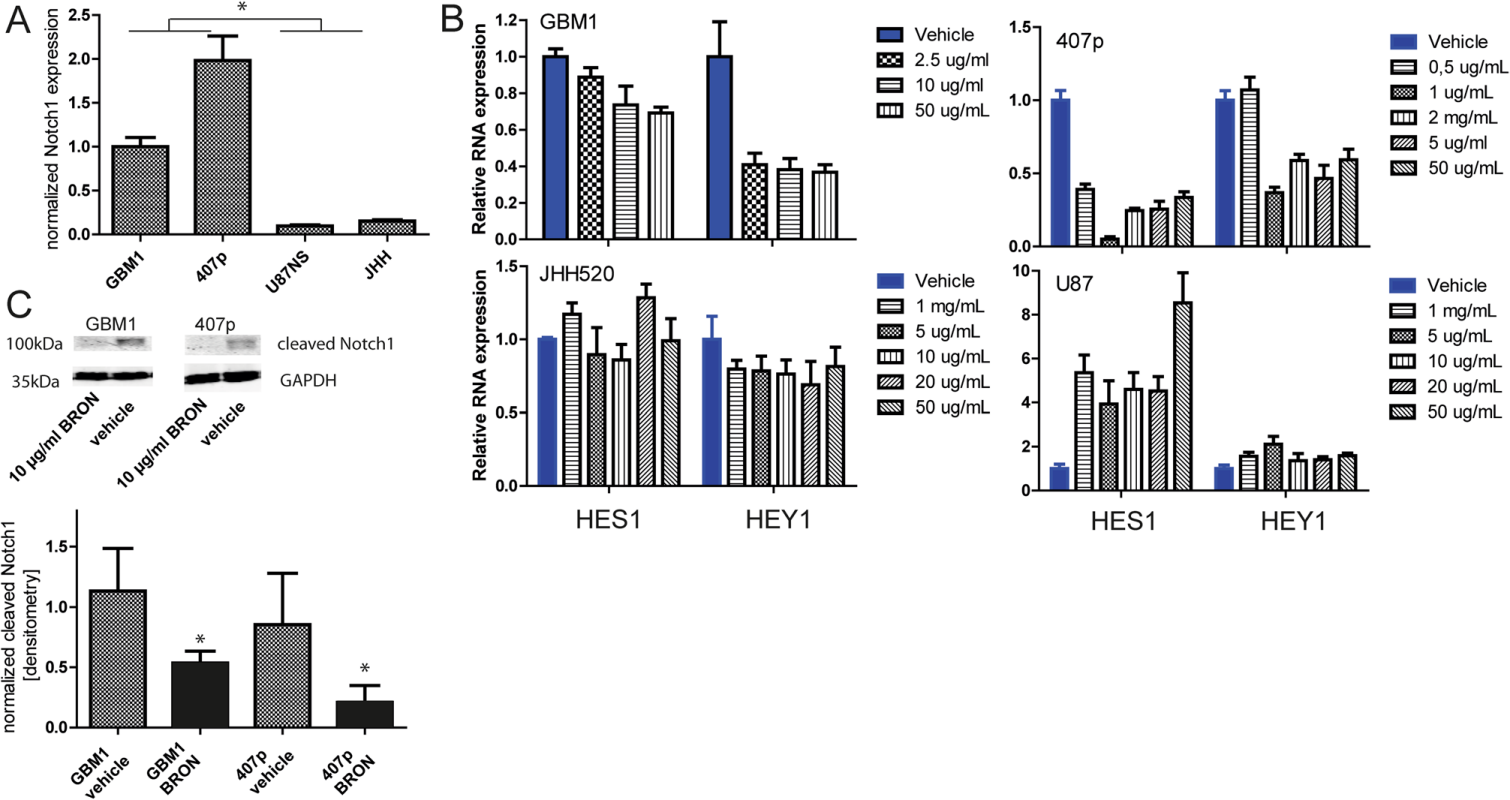
BRON treatment causes suppression of Notch target genes Hes1/Hey1 and cleaved Notch1 receptor protein in GSCs. (**A**) GSCs representing different activation levels of Notch1. (**B**) Identification of drug resistance in our cell models by RT-qPCR based quantification of suppression of Notch pathway targets (HES1 and HEY1) stratified our in vitro platform in responder (GBM1 and 407p) and non-responder (U87 and JHH) cells. We designated minimal drug concentration needed to achieve solid target gene suppression as 10 µg/ml for GBM1 and 1 µg/ml for 407p. (**C**) BRON treatment for 48 h caused significant reduction of cleaved Notch1 receptor protein in responder cells as compared to control/vehicle treatment condition.

By assessing transcript level we found that the glioblastoma models GBM1 and 407p have elevated Notch1 as compared to cell lines U87NS and JHH (Fig. 4A). Spontaneous Notch1 abundancy in our cell models associates with amount of reduction of Hes1 and Hey1 mRNA levels in response to brontictuzumab (BRON) treatment. Our group and others previously established this biomarker signature to be a strong and sensitive reporter for Notch pathway activation in those cell models^28, 31, 43^. Thus, we stratify our cell models in responder (GBM1 and 407p) and non-responder U87NS and JHH as shown by IC_50_ assays (Fig. 4B). Repetitions with selected IC50 have been performed and shown in Figure S2. Since Hes1/Hey are not exclusively regulated by Notch signaling, we performed quantification of cleaved Notch1 receptor as a direct measure to score pathway activity. BRON treatment caused significant suppression of cleaved Notch1 protein in GSCs as compared to control treatment condition (Fig. 4C). Of note, unlike reported regarding the sensitivity of glioma cells to GSI to, treatment response to BRON is not associated to p53 wildtype status. Responder cells coding locus of p53 is mutated (GBM1: exon8: c.832C>T:p.Pro278Ser,exon5:c.388C>A:p.Leu130Ile; 407p: exon5:c.388C>A:p.Leu130Ile, exon6:c.560G>A:p.Gly187Asp, exon8:c.832C>T:p.Pro278Ser; sequencing data can be made available upon request) and non-responder U87 is p53 wildtype^44^. Also, cells that show resistance against BRON are also relatively resistant against GSI MRK003, whereas responder-types reduce Notch pathway target gene expression Hes1/Hey1 in response to MRK003 treatment (Figure S2).

This data shows that high Notch1 activation, and the amount of suppression of Hey1/Hes1 mRNA and cleaved Notch1 protein upon treatment serve as predictive for resistance of GSCs against BRON. Focusing on responder cells, we applied a variety of functional tools to investigate the biological relevance of our observation.

### Brontictuzumab impairs cellular invasion

**Figure 5:**
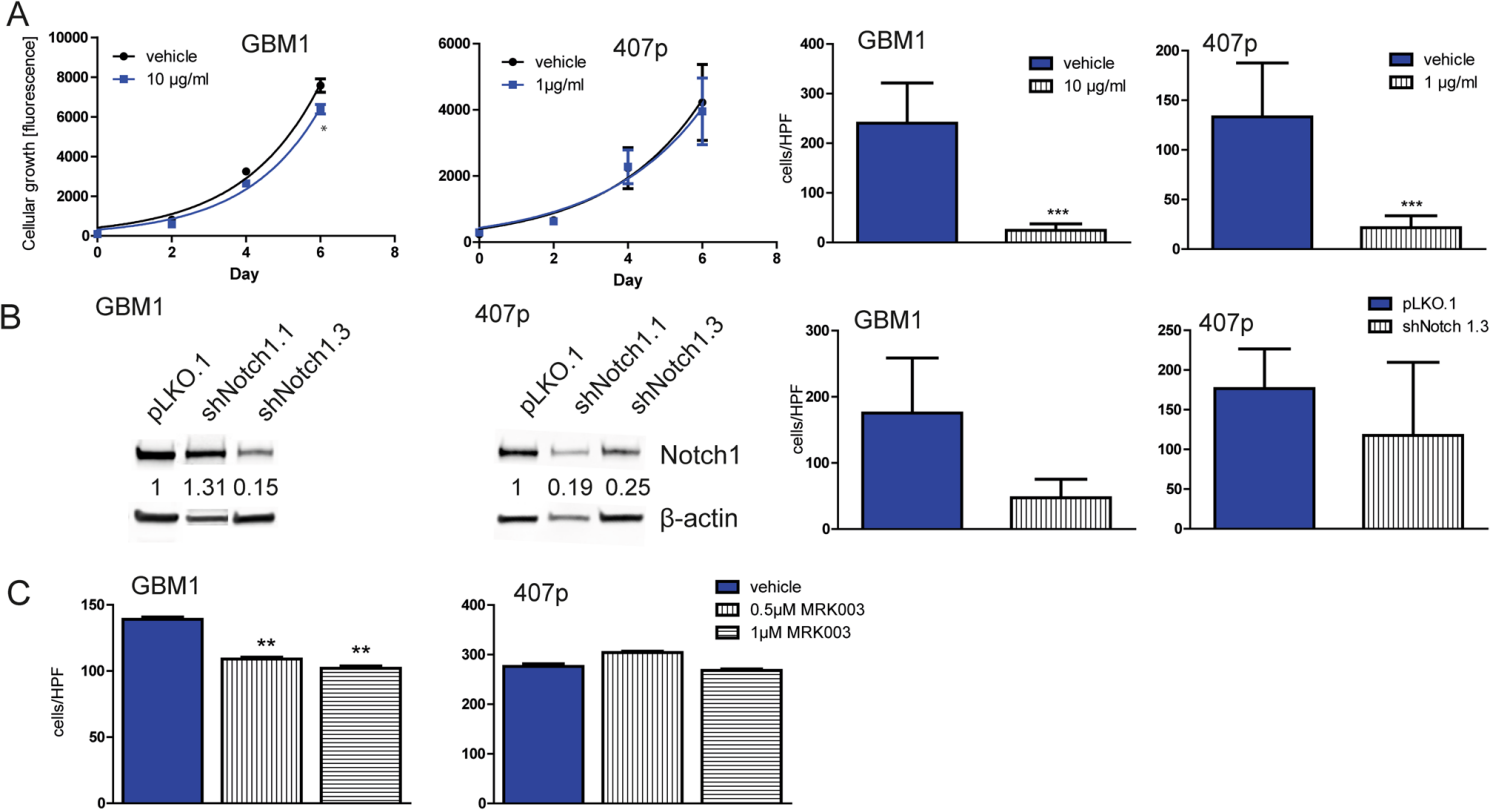
Phenotypic characterization of BRON treatment. (**A**) Mildly (GBM1) or no (407p) effect on cell growth was observed whereas significant reduction in cellular invasion was observed upon long-term exposure to the drug (day 6 treatment). (**B**) Genetic suppression of Notch1 by shRNA did only in part pheno-copied the anti-invasive effect of BRON treatment. (**C**) Exposure of the cells to GSI MRK003 led to reduction of cell invasion in GBM1, which was severely lower as observed under BRON treatment, and no effect in 407p. Validation of previously established MRK003 treatment protocol^28, 43^ see Suppl. Figure S2. Scan of original Western blot membrane can be found in Suppl. Figure S8.

Chronic incubation of the cells to brontictuzumab caused almost no effect on cell growth (Fig. 5A) but significant reduction of cellular invasion (Fig. 5B, day 6 of treatment). We benchmarked the observed anti-invasive effect of BRON to the phenotype the cells show when genetically blocking Notch1 (Fig. 5B) or treating them with MRK003 (Fig. 5C). Both comparator Notch1 conditions did not reach the effects size of BRON therapy. A similar effect on cell growth as observed under BRON treatment was noticed when genetically blocking Notch1 (Suppl. Figure S4).

### Off-target risk of Brontictuzumab

**Figure 6:**
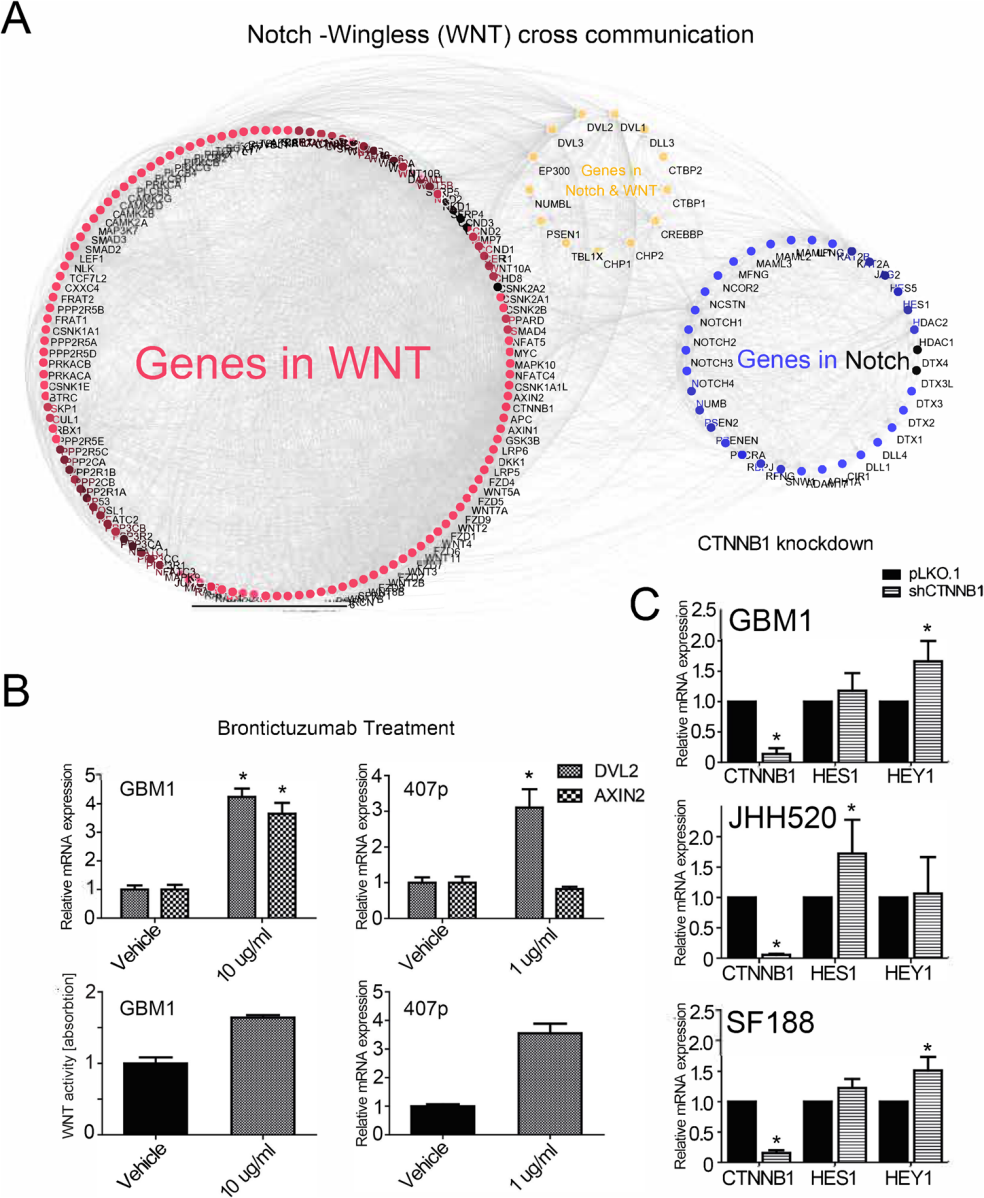
Upregulation of WNT signals in response to BRON treatment. (**A**) Proteins in Notch and WNT pathways showed extensive interactions as revealed by co-occurrence of target genes in both pathways. Red dots represent proteins of the WNT network whereas blue dots represent members of the Notch signaling cascades. Yellow dots represented proteins in both Notch and WNT branch. (**B**) BRON treatment caused activation of expression of consensus WNT target genes (AXIN2, DVL2) and pathway reporter signals in GSCs. (**C**) Genetic suppression of WNT signaling with RNA interference against transcriptional pathway signal mediator beta-catenin (CTNNB1) caused induction of Hes1/Hey1 transcripts.

Since the results with cells with genetic Notch1 blockade, as well as the observed reduction of Hes1/Hey1 mRNA under MRK003 while only showing moderate reduction in cell invasion, let us speculate that the BRON effects are not only due to target suppression. Thus, we performed further off target characterization of the cells under antibody therapy. It is hypothesized that stem cell signaling pathways communicate amongst each other to control stem cell biology in tissues^45^. Protein interaction network analysis found that there is a close direct and indirect interaction between proteins in the Notch and WNT pathways (Fig. 6A). Testing canonical WNT activity by quantifying the transcription of validated WNT pathway target genes^27^ (we chose DVL2 and AXIN2 as our WNT readout marker since previous work suggested their importance in gliomagenesis^33, 46^) , as well as through luciferase WNT reporter signals, we found that the BRON treatment caused upregulation of WNT activity (Fig. 6B). Of note, similar trends of WNT signal activation is observed when treating the cells with MRK003 (Supplementary Figure S7). .Furthermore, activation of Hes1/Hey1 transcript abundancy was noticed when blocking WNT signaling in WNT-dependent^47^ GSC models (Fig. 6C). HPF = high-power field.

## Discussion

The existence of a stem cell population in glioblastoma is controversially discussed. Although computational work on single cell analytics of clinical specimens suggest that glioblastoma is composed of differentiated cells^2^, recent high profile published work of many others further supported the existence of stem cell-like populations in this disease, being regulated be the microenvironment^48^ or specific metabolic^49, 50^ and cell cycle^51, 52^ programs. Inhibiting Notch pathway activity is a powerful strategy to target GSCs^5, 6^ and in experimental studies it has been shown to improve the efficiency of standard of care therapeutic Temozolomide^53^. GSIs are the clinically most relevant anti-Notch drugs^54^, however, the overall success in clinical trials in oncology is rather disappointing due to off-target complications^6, 55^ and emergence of therapy resistance^6^. In a recent trial in glioblastoma patients, the GSI RO4929097 combined with temozolomide and radiotherapy led to the destruction of cells expressing bona fide GSC marker CD133 a putative GSC marker. However, overall clinical success of this treatment may be counteracted due to unwanted effects of induction of mesenchymal differentiation and neo-angiogenesis of the tumors, possibly due to off-target specificity of the drug^56^. Thus, the development of a biomarker signature in glioma tissue predicating the responses to anti-stem cell therapies, and the development of drugs targeting transducers of the Notch signaling activity in this disease other than GSI are of highest clinical need.

By interrogating a hitherto unassessed clinical dataset, our computational framework on transcriptional data revealed that high Notch activity in the glioblastoma is negative prognostic for patients´ median overall survival. Our data also shows that elevated Notch signaling is indicative for stem cell prevalence in the context of brain development and gliomagenesis. Together with existing data, these results further support the plausibility to develop treatment strategies that inhibit Notch activity to improve brain cancer care. Pathway receptor Notch1 is a promising therapeutic target in glioblastoma shown to influence the diseases´ cell survival^9^, growth^10^, motility^11, 12^ , immunogenicity^13^ and tumorigenicity^14^. Notch1 is thought to promote maintenance of GSCs^15–17^ , thereby stress resistance of glioblastoma^18, 19^. However, pharmacological means to exclusively block Notch 1 receptor signaling in glioblastoma are missing. We addressed this by functionally testing BRON, a humanized monoclonal blocking antibody directed against cell surface, signal-receiving domain of Notch1, in stem cell models of the disease. BRON has undergone successful clinical validation in lymphoid malignancies and solid tumors (NCT01703572, NCT01778439)^21, 22, 57^. We now extend the list of tumors in what BRON can inhibit Notch1 receptor signaling pathway and showing therapeutic activity to glioblastoma with phenotypical consequence in impairing cellular invasion. By using MRK003 as the comparator condition representing prominent anti-Notch therapy of GSI, we found this effect is much more prominent. Since the highly infiltrative growth of glioblastoma is the primary cause of the unsatisfying outcomes of surgical intervention, still the clinical treatment option for this disease with the strongest benefit for the patients course^58^, we believe our results propose an interesting drug candidate for further evaluation. Notably, since genetic blockade of Notch1 in our models only in part recapitulates the effect of BRON treatment, possible off-target effects of the antibody have to be acknowledged. Of note, since MRK003 treatment caused a severe suppression in Hes1/Hey1 expression in some of the studied cells, we acknowledge that the proposed biomarkers may only be used to monitor glioma cells´ responsiveness to BRON, but their blockade does not explain the impaired cell invasion. Moreover, induction of Notch target genes in a cell line with low Notch1 activation upon treatment suggests limitations in BRON´s target specificity. As such, we also observed induction of WNT signals upon BRON treatment. Previous reports suggesting a Notch-WNT interaction loop in glioblastoma using similar cell models^11, 31^. We state that the treatment of GSCs with BRON causes activation of WNT signaling by unknown mechanisms, unclear to be caused directly through the chemotherapy/Notch1 signaling blockade or resembles secondary effects. . Given WNT signal activation in glioma cells emerges as a cardinal player in therapy resistance^59^, a follow up study of this observation is highly warranted to validate any counter-therapeutic effect of BRON in glioblastoma. Interestingly, Hes1/Hey1 activation was noticed upon genetic WNT blockage. Since Hes1/Hey1 are not exclusively regulated by Notch signaling, further studies are needed to comprehensively characterize BRON off-target risk and any existence of a reciprocal Notch-WNT interaction in this context. As such, testing the phenotype of responder cells with genetically blocked Notch1 under BRON treatment would be helpful to validate target specificity of this drug candidate.

Notch activity is increased in glioblastoma with IDH1^R132H^ and without 1p/19q co-deletion, it would be interesting to examine whether any differential response to BRON exists in disease models carrying IDH1^R132H^ or 1p/19q co-deletion. Due to the challenges of the field in culturing cell models with IDH1^R132H^ or 1p/19q co-deletion, we focused our analysis on cells with differences in Notch1 expression (all the available and in this study used cell models are IDH1Wt and without 1p/19 co-deletion, data not shown). Cells with higher Notch1 protein levels are more sensitive to BRON, suggesting Notch1 can serve as a biomarker for treatment resistance. Our observation is in line with previous data showing that high Notch signals in glioblastoma predicts for sensitivity to pan-Notch inhibitors GSI^42^. Of note, BRON sensitivity seems not to be associated to wildtype p53 status, which is different to recent observations on sensitivity towards GSI^60^. We acknowledge that more cell models should be tested to further validate this hypothesis.

## Conclusion

Notch signaling activity related biomarker signatures is useful to predict stem cell prevalence in neural context, to predict the overall survival outlook of patients with glioblastoma as well as to predict therapy resistance of the tumor towards anti-Notch directed drugs. Notch pathway activity thus may help to guide the development of novel precision treatment option in glioblastoma. BRON is a promising drug candidate to block receptor specific Notch activity in glioblastoma cells that can help to tackle the hitherto unresolved problem of tumor cell invasion. However, further studies to delineate BRON´s therapeutic potential in animal studies and characterization of its off-target risk and underlying mode of action leading to cell invasion blockade are needed.

## Supplementary information

Supplementary Legends.

Supplementary Table S1.

Supplementary Table S2.

Supplementary Table S3.

Supplementary Table S4.

Supplementary Table S5.

Supplementary Figures

## Data Availability

The datasets used and/or analyzed during the study are available from the corresponding author on reasonable request.

## Abbreviations

BRON: Brontictuzumab
GBM: Glioblastoma
GSC: Glioma stem-like cell
GSI: Gamma-secretase inhibitor
IDH: Isocytrate dehydrogenase
WNT: Wingless
