# Supplementary material for: A computational guided, functional validation of a novel therapeutic antibody proposes Notch signaling as a clinical relevant and druggable target in glioma
Source: Sci Rep. 2020 Oct 1;10:16218. doi: 10.1038/s41598-020-72480-y (PMC7531005; doi:10.1038/s41598-020-72480-y)
Supplement: Supplementary file 1 — Supplementary Legends. [file 41598_2020_72480_MOESM1_ESM.docx]

**Supplementary data files:**

Figure S1: Primer sequences and antibodies used in the study.

Figure S2: Confirmatory responses of Notch target gene expression in GBM1 and 407p under BRON or MRK003 treatment.

Figure S3: Comparison of KEGG item enrichment in glioma stem-like cells compared to differentiated counterparts. Listing of KEGG items in Table S5.

Figure S4: Cellular growth of GBM1 and 407p with genetic reduced Notch1 activation and their respective empty vector control.

Figure S5: Comparison of mutational burden of gliomas with high vs. low Notch activation score

Figure S6: Details on molecular association of gliomas with high vs. low Notch activation score

Figure S7: Effects of MRK003 treatment on WNT pathway activation signals in glioma cells GBM1 and 407p (WNT reporter assay and WNT pathway target genes)

Figure S8: Original Western blot membranes on genetic suppression of Notch1 used in this results presentation

Figure S9: Original Western blot membrane on inhibition of cleaved Notch1 (48 hours treatment with BRON, concentration 10µg/ml) used in this results presentation.

Table S1: Clinical information and quantity of patients in our cohorts.

Table S2: Details on the computation on the association of Notch pathway target genes to our clinical sample Notch score

Table S3: Details on the computation on KEGG items upregulated in neural stem cells compared to astrocytes.

Table S4: Details on the computation on KEGG items and their clinical prognostic value in glioma patients.

Table S5: Details on the computation on KEGG items in glioma stem cells and differentiated counterparts.
