# Supplementary figures and images for: A computational guided, functional validation of a novel therapeutic antibody proposes Notch signaling as a clinical relevant and druggable target in glioma
Source: Sci Rep. 2020 Oct 1;10:16218. doi: 10.1038/s41598-020-72480-y (PMC7531005; doi:10.1038/s41598-020-72480-y)

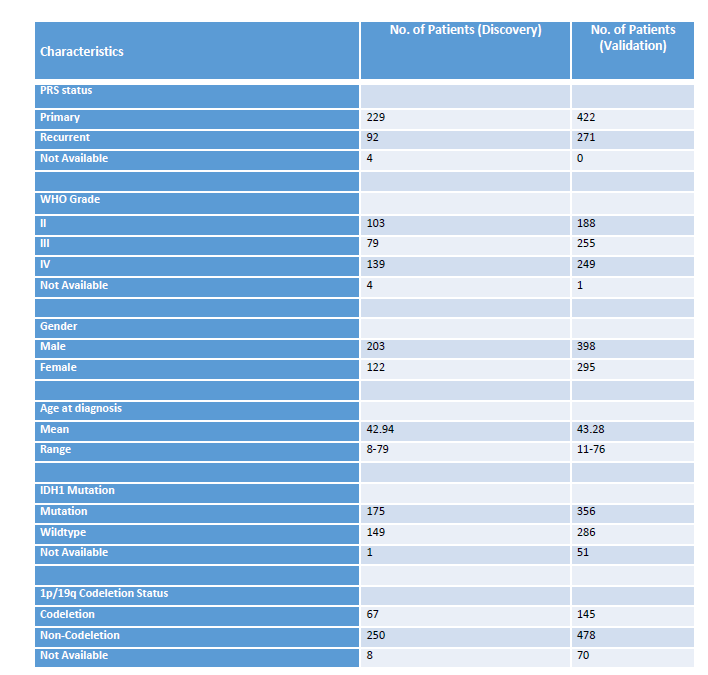

Supplement: Supplementary file 2 — Supplementary Table S1. [file 41598_2020_72480_MOESM2_ESM.tif]
