# Supplementary material for: A computational guided, functional validation of a novel therapeutic antibody proposes Notch signaling as a clinical relevant and druggable target in glioma
Source: Sci Rep. 2020 Oct 1;10:16218. doi: 10.1038/s41598-020-72480-y (PMC7531005; doi:10.1038/s41598-020-72480-y)
Supplement: Supplementary file 7 — Supplementary Figures [file 41598_2020_72480_MOESM7_ESM.pptx]

## Slide 1
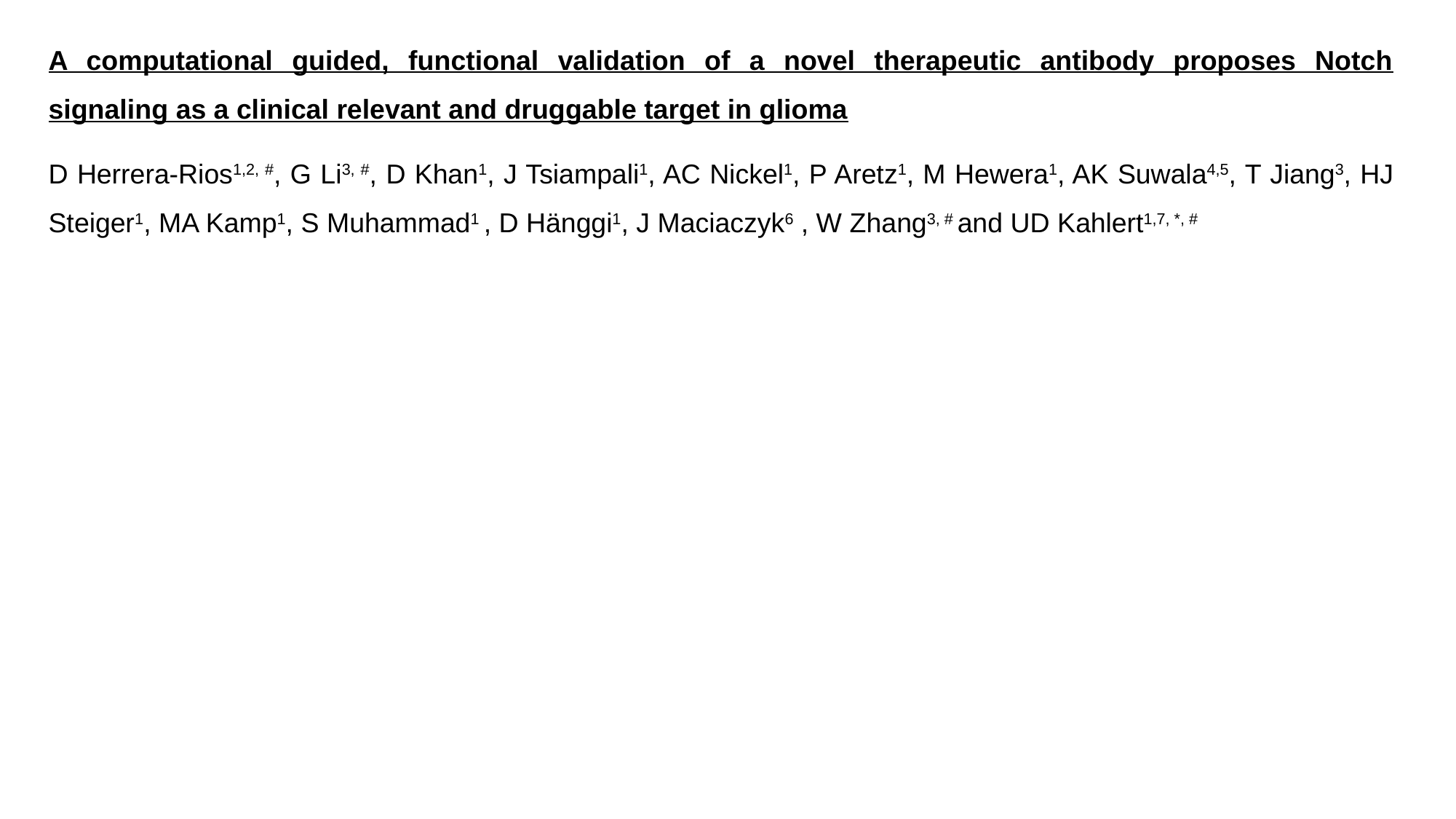

A computational guided, functional validation of a novel therapeutic antibody proposes Notch signaling as a clinical relevant and druggable target in glioma
D Herrera-Rios1,2, #, G Li3, #, D Khan1, J Tsiampali1, AC Nickel1, P Aretz1, M Hewera1, AK Suwala4,5, T Jiang3, HJ Steiger1, MA Kamp1, S Muhammad1 , D Hänggi1, J Maciaczyk6 , W Zhang3, # and UD Kahlert1,7, *, #

## Slide 2
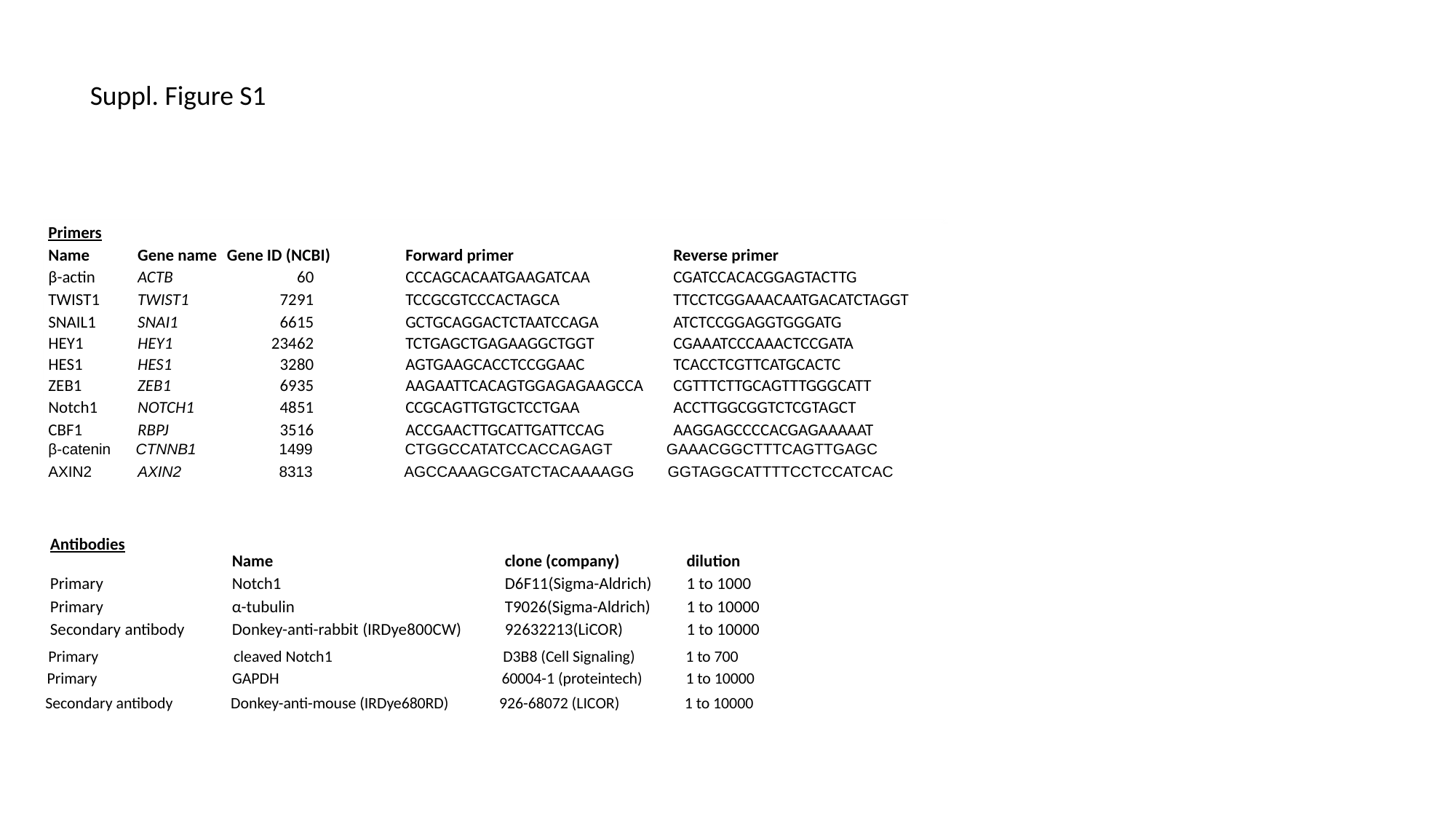

Suppl. Figure S1
| Primers | | | | | | | | | |
| --- | --- | --- | --- | --- | --- | --- | --- | --- | --- |
| Name | Gene name | Gene ID (NCBI) | | Forward primer | | | Reverse primer | | |
| β-actin | ACTB | 60 | | CCCAGCACAATGAAGATCAA | | | CGATCCACACGGAGTACTTG | | |
| TWIST1 | TWIST1 | 7291 | | TCCGCGTCCCACTAGCA | | | TTCCTCGGAAACAATGACATCTAGGT | | |
| SNAIL1 | SNAI1 | 6615 | | GCTGCAGGACTCTAATCCAGA | | | ATCTCCGGAGGTGGGATG | | |
| HEY1 | HEY1 | 23462 | | TCTGAGCTGAGAAGGCTGGT | | | CGAAATCCCAAACTCCGATA | | |
| HES1 | HES1 | 3280 | | AGTGAAGCACCTCCGGAAC | | | TCACCTCGTTCATGCACTC | | |
| ZEB1 | ZEB1 | 6935 | | AAGAATTCACAGTGGAGAGAAGCCA | | | CGTTTCTTGCAGTTTGGGCATT | | |
| Notch1 | NOTCH1 | 4851 | | CCGCAGTTGTGCTCCTGAA | | | ACCTTGGCGGTCTCGTAGCT | | |
| CBF1 | RBPJ | 3516 | | ACCGAACTTGCATTGATTCCAG | | | AAGGAGCCCCACGAGAAAAAT | | |
β-catenin CTNNB1	 1499	 CTGGCCATATCCACCAGAGT GAAACGGCTTTCAGTTGAGC
AXIN2 AXIN2	 8313	 AGCCAAAGCGATCTACAAAAGG GGTAGGCATTTTCCTCCATCAC
| Antibodies | | Name | | | clone (company) | dilution |
| --- | --- | --- | --- | --- | --- | --- |
| Primary | | Notch1 | | | D6F11(Sigma-Aldrich) | 1 to 1000 |
| Primary | | α-tubulin | | | T9026(Sigma-Aldrich) | 1 to 10000 |
| Secondary antibody | | Donkey-anti-rabbit (IRDye800CW) | | | 92632213(LiCOR) | 1 to 10000 |
Primary	 cleaved Notch1 		 D3B8 (Cell Signaling) 1 to 700
Primary	 GAPDH 		 60004-1 (proteintech) 1 to 10000
Secondary antibody Donkey-anti-mouse (IRDye680RD) 926-68072 (LICOR) 1 to 10000

## Slide 3
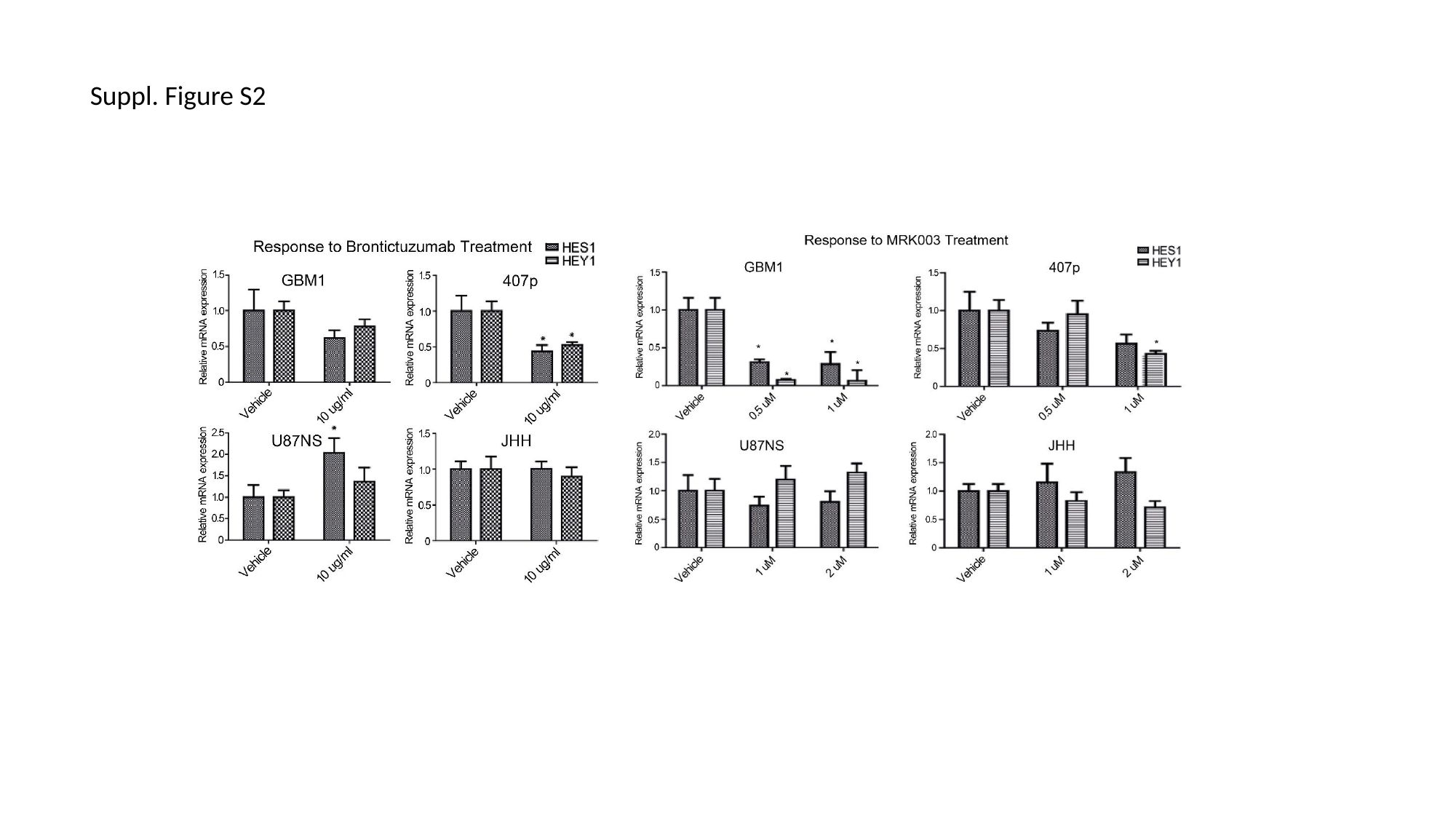

Suppl. Figure S2

## Slide 4
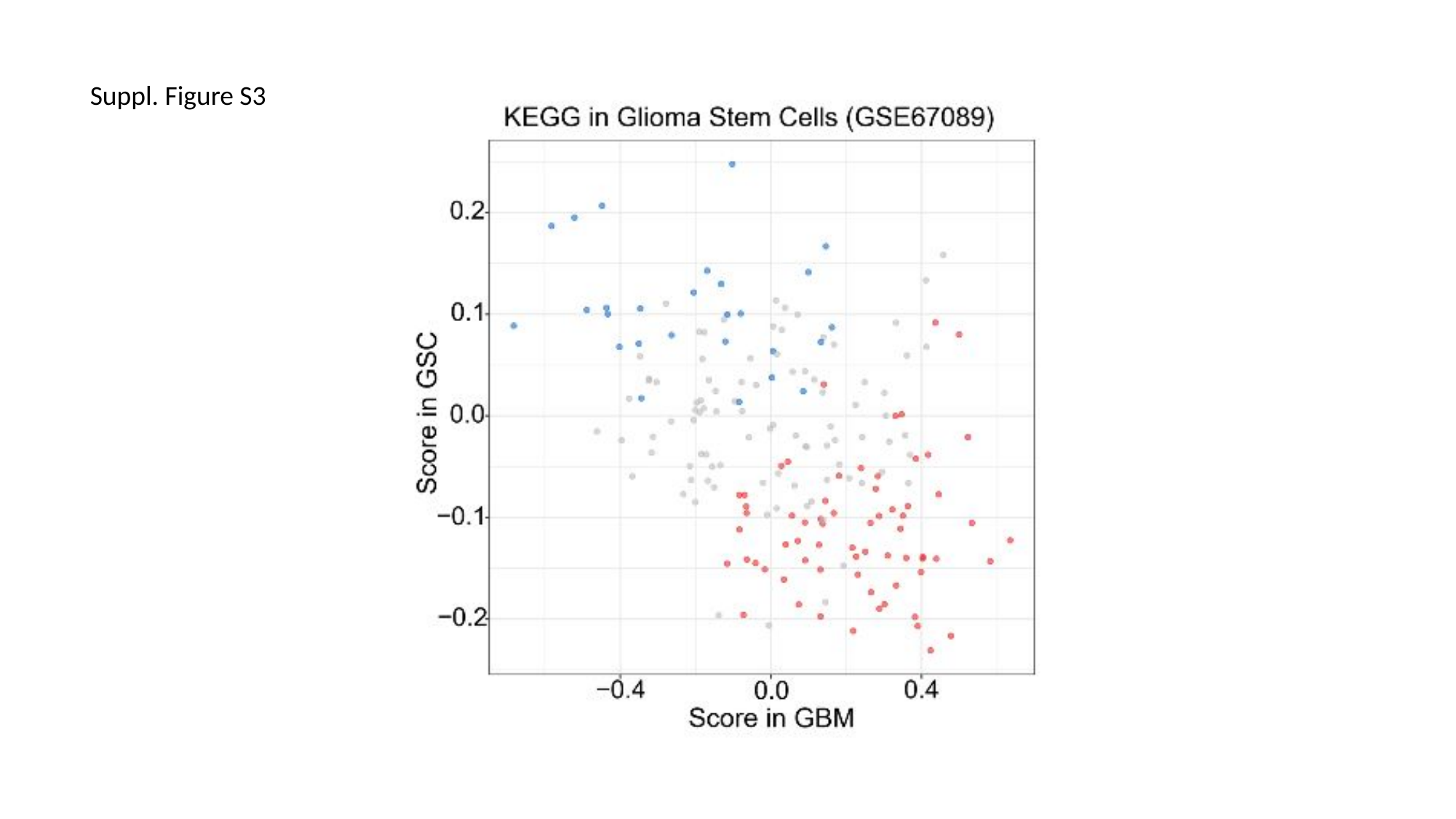

Suppl. Figure S3

## Slide 5
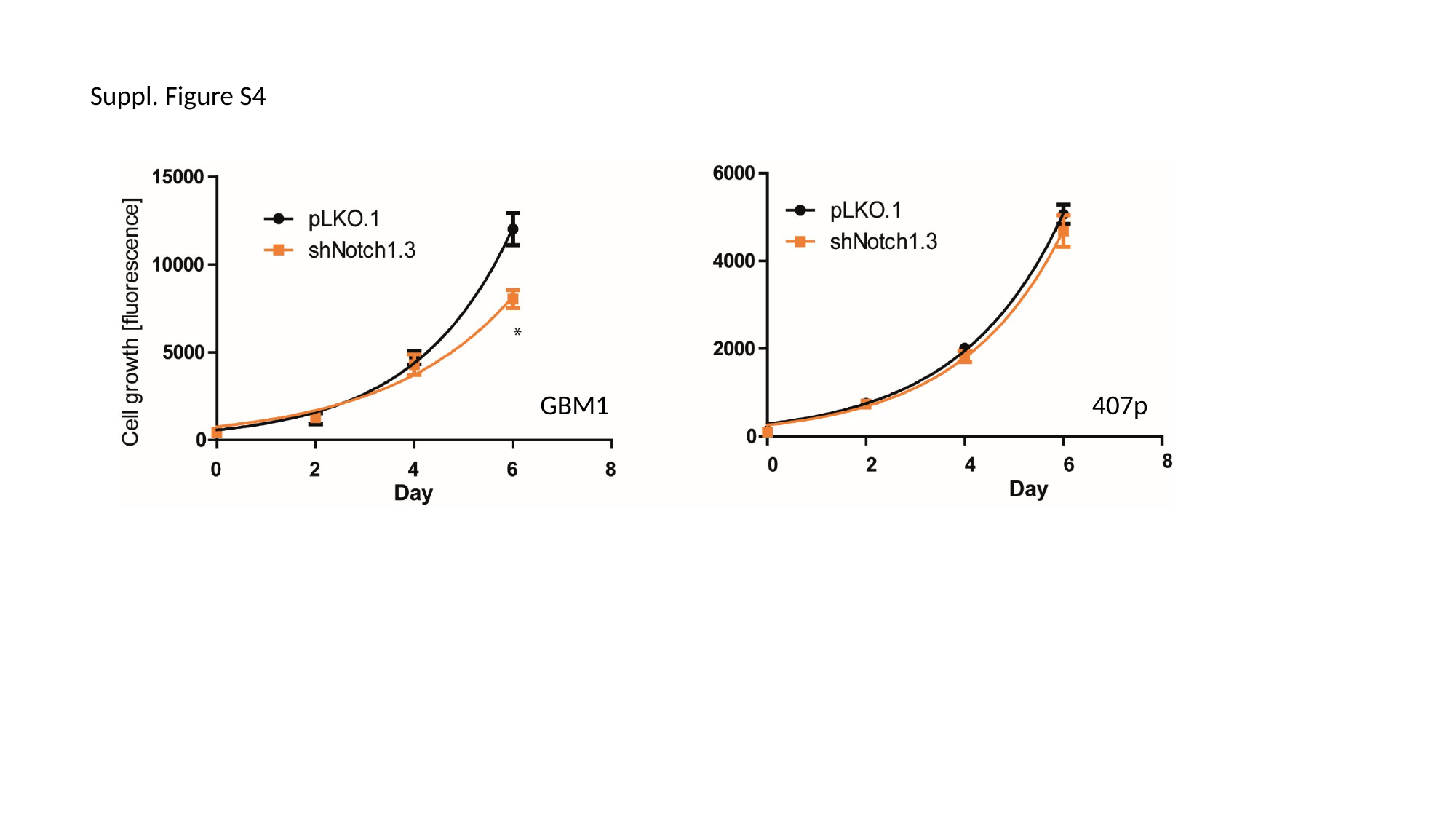

Suppl. Figure S4
GBM1
407p

## Slide 6
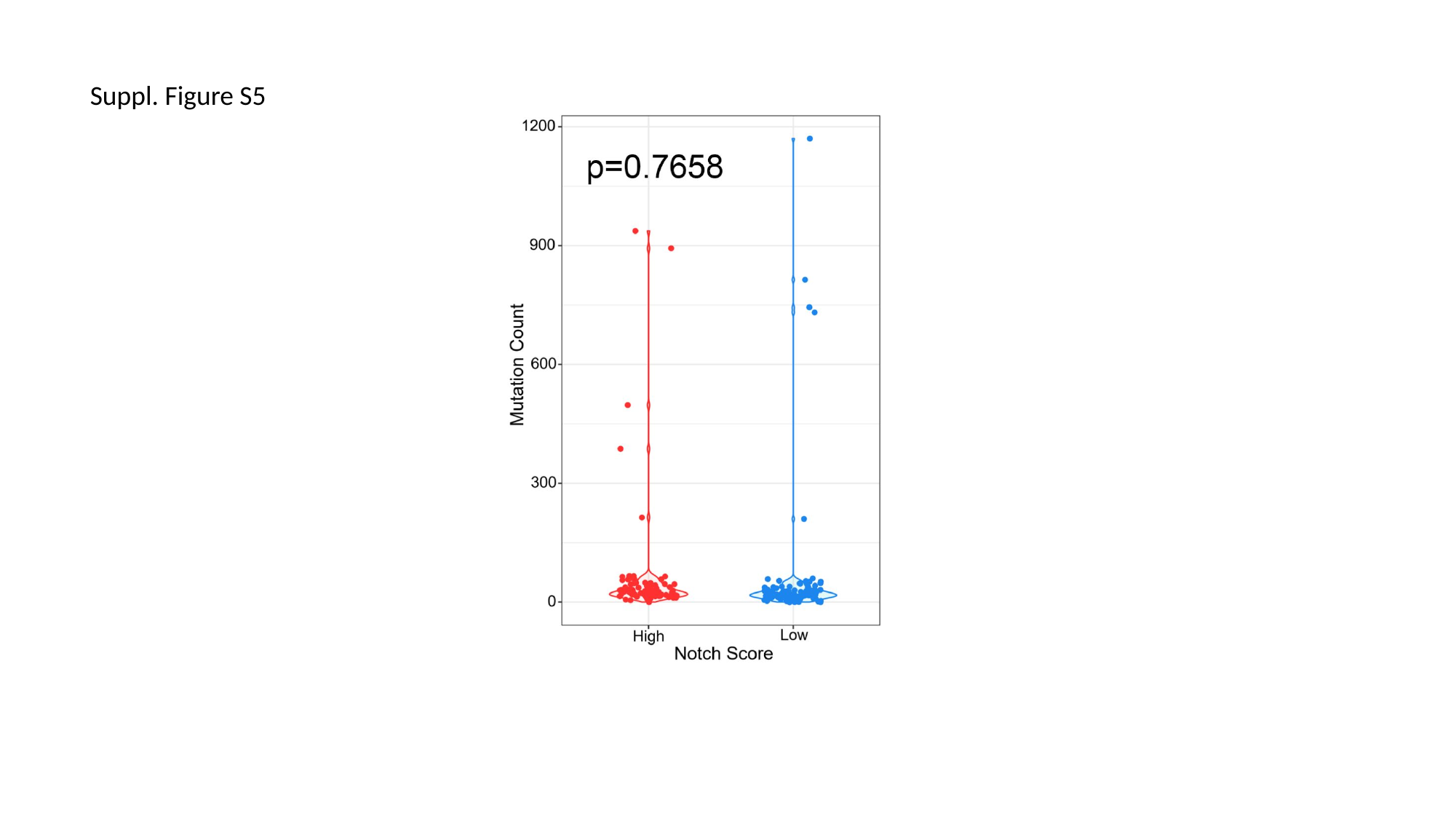

Suppl. Figure S5

## Slide 7
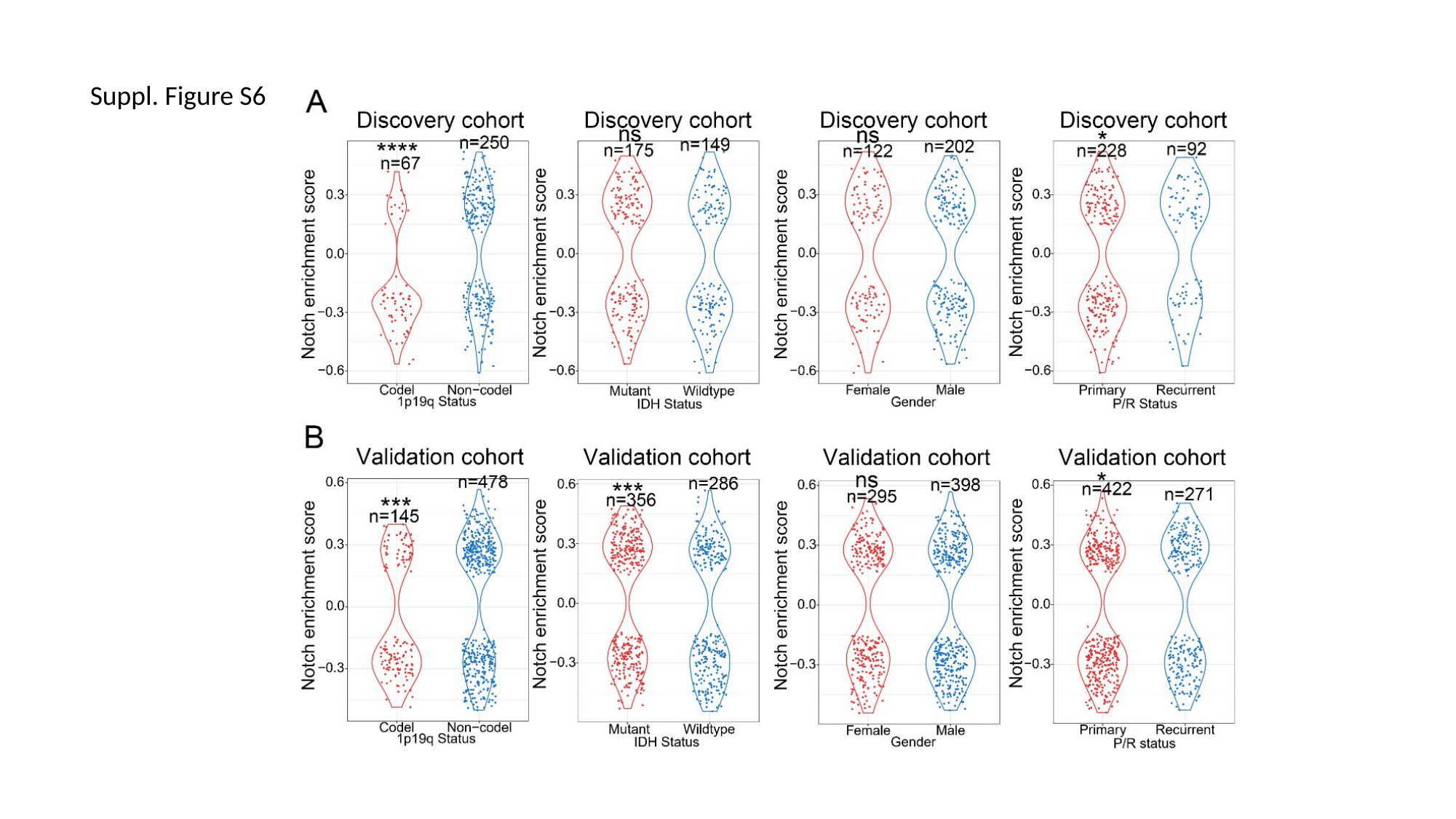

Suppl. Figure S6

## Slide 8
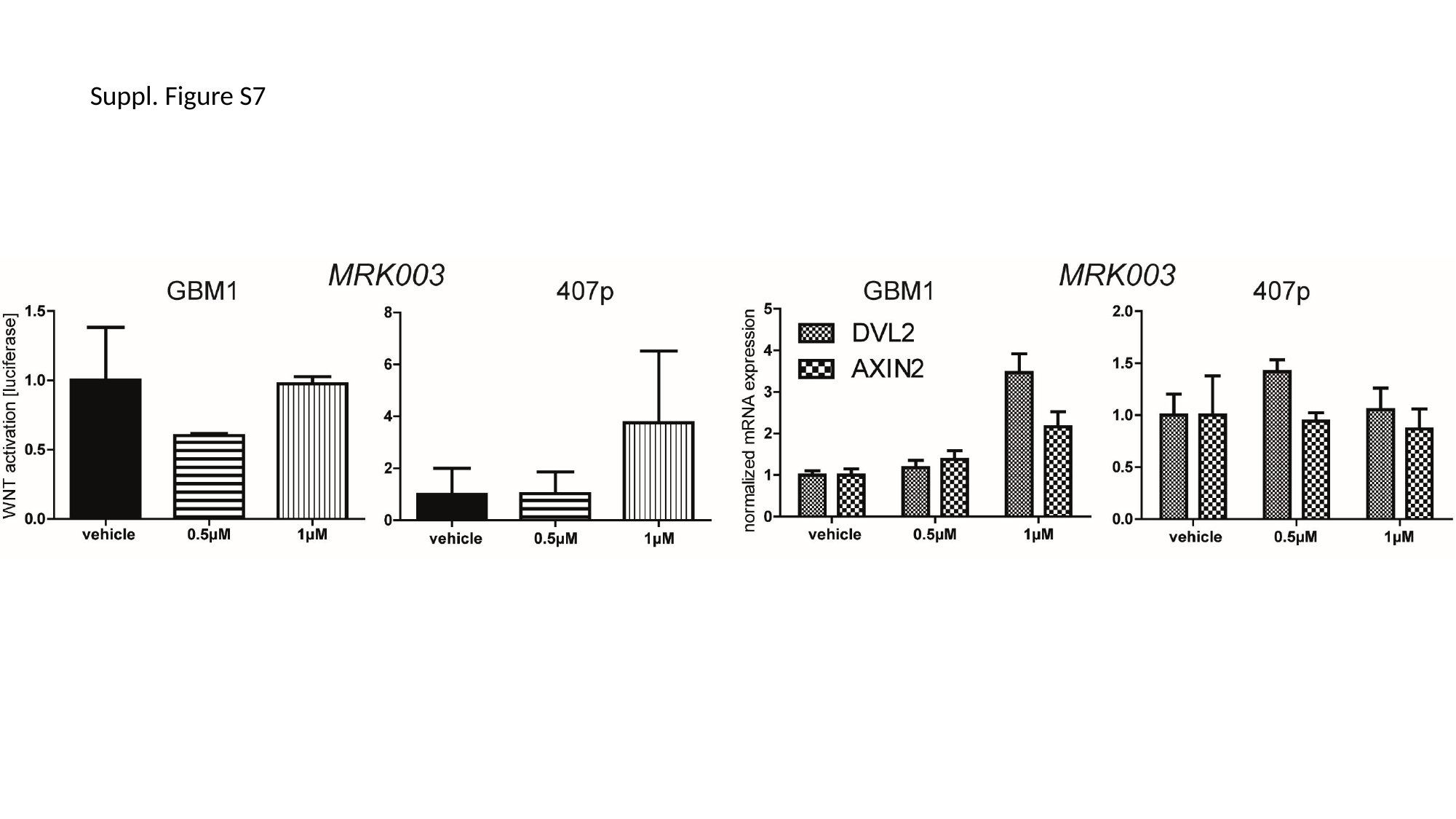

Suppl. Figure S7

## Slide 9
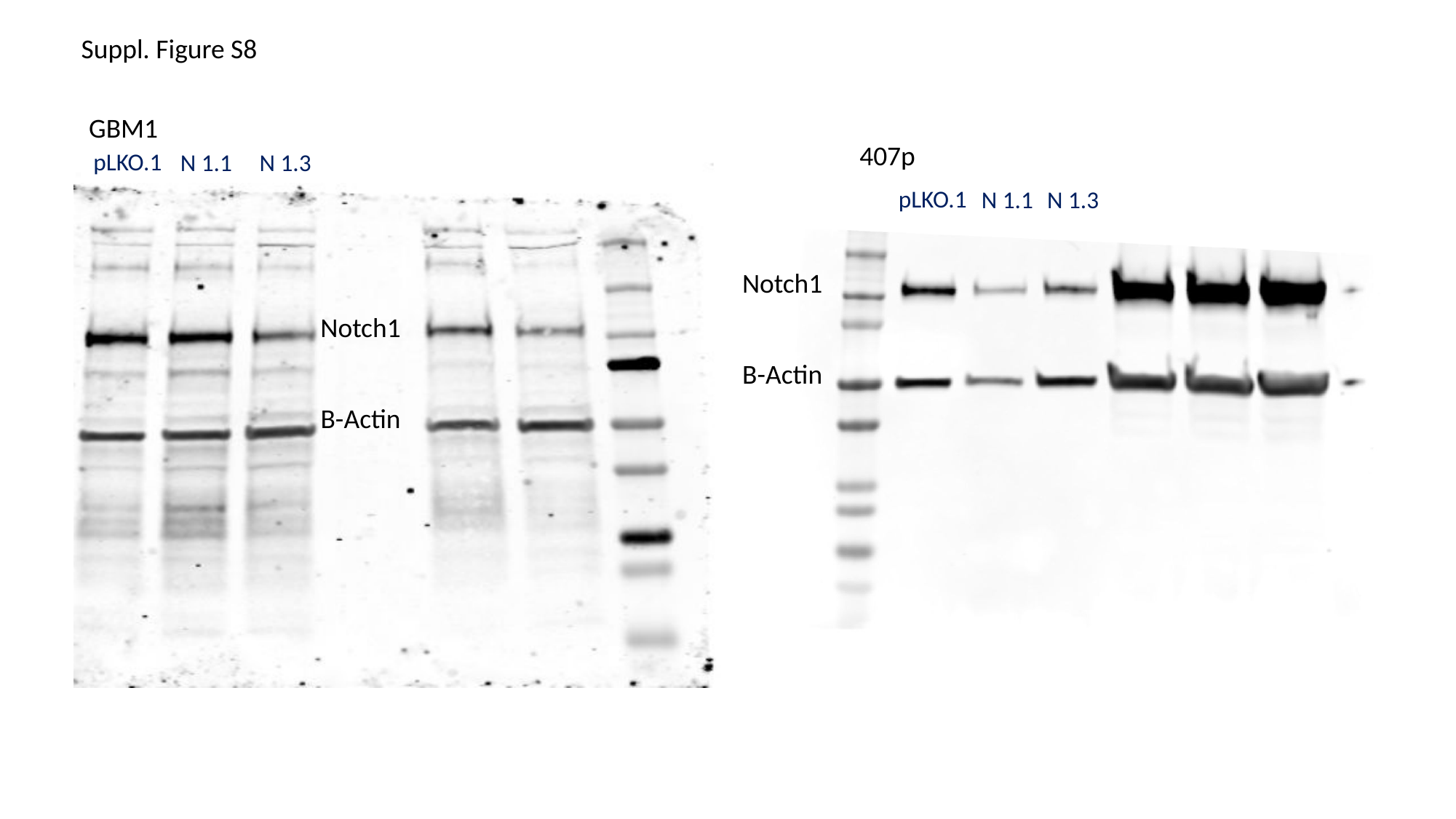

Suppl. Figure S8
GBM1
407p
pLKO.1
N 1.3
N 1.1
pLKO.1
N 1.1
N 1.3
Notch1
Notch1
B-Actin
B-Actin

## Slide 10
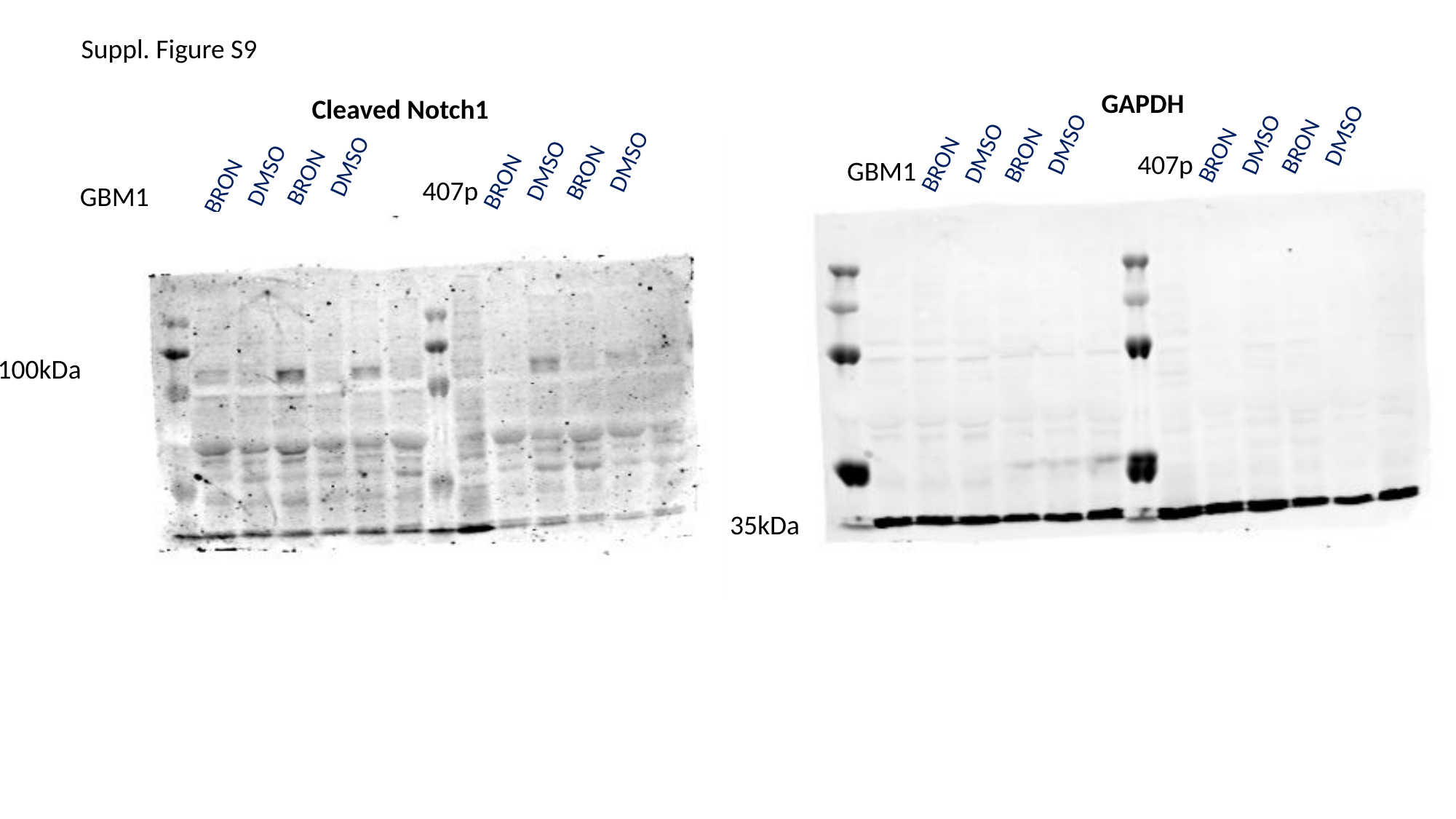

Suppl. Figure S9
DMSO
BRON
DMSO
BRON
407p
GAPDH
DMSO
BRON
DMSO
BRON
GBM1
Cleaved Notch1
DMSO
BRON
DMSO
BRON
407p
DMSO
BRON
DMSO
BRON
GBM1
100kDa
35kDa
GAPDH
